# Artemisinin kills malaria parasites by damaging proteins and inhibiting the proteasome

**DOI:** 10.1038/s41467-018-06221-1

**Published:** 2018-09-18

**Authors:** Jessica L. Bridgford, Stanley C. Xie, Simon A. Cobbold, Charisse Flerida A. Pasaje, Susann Herrmann, Tuo Yang, David L. Gillett, Lawrence R. Dick, Stuart A. Ralph, Con Dogovski, Natalie J. Spillman, Leann Tilley

**Affiliations:** 1 Department of Biochemistry and Molecular Biology, Bio21 Molecular Science and Biotechnology Institute, The University of Melbourne, Melbourne, VIC 3010 Australia; 2 Oncology Clinical R&D, Takeda Pharmaceuticals International Co, Cambridge, MA 02139 USA

## Abstract

Artemisinin and its derivatives (collectively referred to as ARTs) rapidly reduce the parasite burden in *Plasmodium falciparum* infections, and antimalarial control is highly dependent on ART combination therapies (ACTs). Decreased sensitivity to ARTs is emerging, making it critically important to understand the mechanism of action of ARTs. Here we demonstrate that dihydroartemisinin (DHA), the clinically relevant ART, kills parasites via a two-pronged mechanism, causing protein damage, and compromising parasite proteasome function. The consequent accumulation of proteasome substrates, i.e., unfolded/damaged and polyubiquitinated proteins, activates the ER stress response and underpins DHA-mediated killing. Specific inhibitors of the proteasome cause a similar build-up of polyubiquitinated proteins, leading to parasite killing. Blocking protein synthesis with a translation inhibitor or inhibiting the ubiquitin-activating enzyme, E1, reduces the level of damaged, polyubiquitinated proteins, alleviates the stress response, and dramatically antagonizes DHA activity.

## Abstract (web summary)

Artemisinin (ART) is a widely used antimalarial drug, but its mechanism of action is poorly understood. Here, Bridgford et al. show that ART kills parasites by a two-pronged mechanism, causing protein damage and compromising proteasome function, and that accumulation of proteasome substrates activates the ER stress response.

## Introduction

Malaria remains a scourge of humanity, affecting hundreds of millions of people and causing ~440,000 deaths each year^1^. Current antimalarial control is highly dependent on artemisinin (ART) combination therapies (ACTs). Thus, it is very concerning that parasites with decreased sensitivity to this drug class have emerged in South East Asia, delaying the clearance of parasites from patients^2^. In areas with concomitant partner drug resistance, up to ~50% treatment failure is now observed, and the decreased efficacy of ARTs is putting further pressure on the partner drugs^3, 4^. Even more worryingly, the first indigenous African parasite isolate with mutations in the resistance marker (*K13*) has been reported^5^. Thus, it is critically important to understand the mechanism of action of ARTs, with a view to overcoming resistance.

DHA is known to cause widespread damage to parasite proteins in different cellular compartments^6–8^. These damaged proteins would be expected to be targeted for ubiquitination and degradation by the proteasome; and changes that affect this pathway might be expected to help enable parasites to survive DHA exposure.

Mutations in a Kelch domain protein (K13-propeller; PF3D7_1343700) are associated with decreased ART sensitivity in vitro;^9^ and genetic manipulation of the *K13* locus has confirmed a role in the decreased sensitivity phenotype, although other genetic factors also contribute^10^. K13 shows sequence similarity with a class of substrate adaptors that facilitate ubiquitin ligation^9^, suggesting that protein ubiquitination plays a role in ART action or resistance^11^ and mutations in ubiquitination machinery have been reported to be associated with ART resistance^12^. Indeed, we have previously shown that proteasome inhibitors act synergistically with ARTs^13^, further implicating the ubiquitin/proteasome pathway in ART action and resistance. Until now, however, the molecular details have remained unclear.

Here we identify proteasome function as a key target of DHA and pinpoint the build-up of polyubiquitinated proteins and the consequent endoplasmic reticulum (ER) stress response as the key toxic events. We show that inhibition of protein polyubiquitination protects parasites against DHA-mediated killing. The work reveals new points of vulnerability that could be targeted with chemotherapies against malaria parasites.

## Results

### DHA treatment induces an ER stress response

**Fig. 1:**
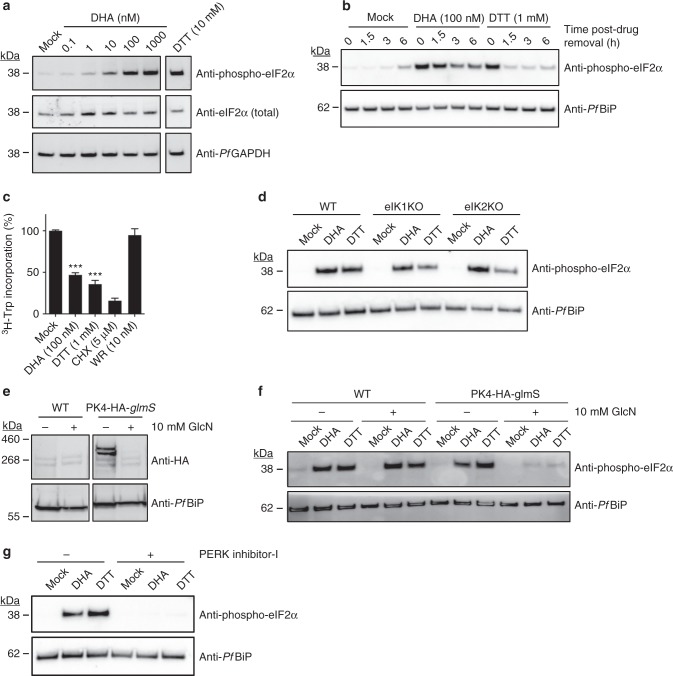
DHA activates an unfolded protein response, mediated by PK4. **a**, **b** Trophozoites (**a** 3D7, **b** Cam3.II_rev) were treated with 0.1% DMSO (mock), DHA or DTT for 90 min and harvested immediately (**a**) or washed and returned to culture for the indicated amount of time (**b**) before lysates were subjected to Western blot analysis and membranes probed for phosphorylated-eIF2α. **c** Trophozoites (Cam3.II_rev) were incubated with indicated compounds for 1 h and protein synthesis measured. Error bars represent s.e.m. ***P < 0.001 between mock and DHA or DTT treated samples (*n* *=* 5 (Mock); *n* *=* 4 (DTT); *n* *=* 3 (DHA, WR99210, CHX)), ANOVA. **d** *eIK1* and *eIK2* knock-out trophozoite-stage parasites (in a 3D7 background) were treated with 0.1% DMSO (mock), 1 μM DHA or 1 mM DTT for 60 min and lysates were subjected to Western blot analysis and probed for phosphorylated-eIF2α. **e**, **f** *PK4-HA-glmS* (in a 3D7 background) or *WT* (3D7) trophozoite-infected RBCs were treated with 10 mM glucosamine (GlcN) from the ring stage of the previous cycle and lysates were subjected to Western blot analysis, probing with anti-HA (**e**), or parasites were treated with 0.1% DMSO (mock), 100 nM DHA or 1 mM DTT for 60 min and lysates were subjected to Western blot analysis, probing for phosphorylated eIF2α (**f**). **g** Trophozoites (Cam3.II_rev) were treated with 20 μM PERK Inhibitor-I for 3 h, followed by a further hour in 0.1% DMSO (mock), 1 μM DHA or 1 mM DTT and lysates were analysed by Western blot for phosphorylated-eIF2α. Loading controls, *Pf*GAPDH or *Pf*BiP. All blots are representative of at least three independent experiments. Additional details in Supplementary Fig. 1

We previously reported that rings and early trophozoites exhibit growth retardation following exposure to very short (sub-lethal) pulses of DHA^13^. This result suggested DHA may initiate a stress response, so we examined the ER stress response hallmarks, eIF2α phosphorylation and translational arrest. We subjected ART-sensitive (*K13* wildtype, 3D7 line) trophozoites to pulsed exposure to DHA (60–90 min), an exposure duration designed to reflect the short (~1 h) in vivo half-life of DHA^14^. We found that eIF2α is phosphorylated in a concentration-dependent manner upon exposure to DHA (Fig. 1a). A similar response was observed in mid-ring stage (Supplementary Fig. 1a). The level of phosphorylation is similar to that observed with the known inducer of ER stress, dithiothreitol (DTT; Fig. 1a). By contrast, exposure to 1 μM chloroquine (60 min), which has a slower onset of killing^15^, did not induce eIF2α phosphorylation (Supplementary Fig. 1b). Upon removal of DTT, eIF2α phosphorylation is rapidly reversed, indicating recovery from the stress, whereas DHA-induced phosphorylation is still evident 6 h later (Fig. 1b), indicating prolonged ER stress.

Consistent with ER stress slowing (but not arresting) protein translation^16^, we found that parasite protein synthesis was decreased by 65% in the presence of DTT, while cycloheximide (CHX) caused more complete inhibition (Fig. 1c). Similarly, exposure to 100 nM DHA (1 h) reduced protein translation by ~ 50% (Fig. 1c). By contrast, a lethal pulse of the antifolate antimalarial, WR99210 which exhibits a slow onset of killing^15^ (see Supplementary Fig. 1c for kill curves), had no immediate effect on protein translation. The partial arrest of translation was indicated by lack of complete inhibition, even at higher DHA concentrations (Supplementary Fig. 1d).

### PK4 is responsible for the DHA-initiated ER stress response

To determine the kinase responsible for eIF2α phosphorylation, we examined the effects of genetically modifying the three eIF2α kinases, eIK1, eIK2 and PK4, that have been identified in *P. falciparum*^17^. eIK2 (which controls the latency of sporozoites)^18^ and, the GCN2 homolog, eIK1 (which regulates eIF2α phosphorylation in response to amino acid starvation)^19^ have been shown to be dispensable in *P. falciparum*^20^. We found that eIF2α is still phosphorylated following DHA and DTT treatment in *eIK1*- or *eIK2*-knock-out lines (Fig. 1d), indicating that they are not responsible for DHA-induced eIF2α phosphorylation.

The PERK homologue, PK4, has been reported to control global protein translation levels in late schizonts, and to be essential for growth^20, 21^. We generated a hemagglutinin (HA)-tagged inducible knockdown transgenic parasite line, *PK4* mRNA fused to the *glmS* riboswitch^22^. Upon the addition of glucosamine (GlcN), the *glmS* ribozyme self-cleaves at the 3′ UTR and the target mRNA is degraded. As anticipated, upon addition of 10 mM GlcN, marked depletion of PK4-HA was observed (Fig. 1e). While 10 mM GlcN had no effect on eIF2α phosphorylation levels in the 3D7 parent, it ablated DHA/DTT-induced eIF2α phosphorylation in the *PK4* knock-down line. These data are consistent with PK4 being the kinase responsible for eIF2α phosphorylation, and further validates the suggestion that DHA initiates an unfolded protein stress response in *P. falciparum*. To further investigate the role of PK4, we made use of a potent cell-permeable, inhibitor of mammalian PERK ((PERK Inhibitor-I or GSK2606414)^23^). Exposure of *P. falciparum* trophozoites to PERK Inhibitor-I (20 μM) for 4 h had no effect on parasite development; however, DHA/DTT-induced eIF2α phosphorylation was prevented (Fig. 1g), again supporting the role of this kinase in DHA-induced eIF2α phosphorylation.

### Accumulated polyubiquitinated proteins underpin DHA toxicity

**Fig. 2:**
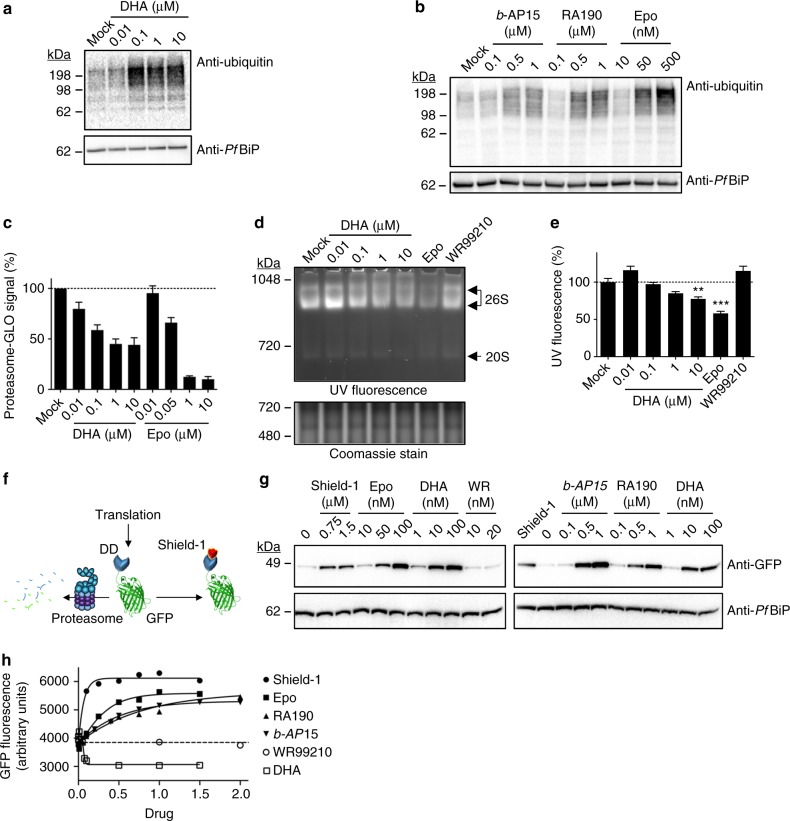
DHA causes a build-up of ubiquitinated protein by inhibiting the proteasome. **a**, **b** Trophozoites (3D7) were incubated with 0.1% DMSO (mock) or DHA (90 min) (**a**) or *b*-AP15, RA190 or Epo (epoxomicin) (3 h) (**b**) and extracts were probed for ubiquitinated proteins. Blots are representative of at least three independent experiments (see Supplementary Fig. 3 for biological replicates). Loading control, *Pf*BiP. **c**, **d** Trophozoites (Cam3.II_rev) were incubated with indicated compounds for 3 h (**c**), or 1 h (**d**), before proteasome-GLO activity (**c**), or non-reducing PAGE proteasome analysis (**d**). Error bars represent s.e.m. (*n* = 3). **e** Quantitation of native gels (data from **d**). Error bars represent s.e.m. **P < 0.01 between mock and 10 μM DHA-treated samples, ***P < 0.001 between mock and epoxomicin treated samples (*n* = 4), ANOVA. **f** Schematic of the GFP-DD reporter system. **g**, **h** GFP-DD transfectants (in a 3D7 background) were maintained in Shield-1 for 24 h, before wash-out. Trophozoites were re-exposed to Shield-1 or indicated compounds for 4 h. **g** Cell extracts were analysed by Western blot for GFP. Blot is representative of at least three independent experiments. Loading control, *Pf*BiP. **h** GFP fluorescence measured by flow cytometry. Concentrations of Shield-1 (closed circles), RA190 (upright triangles), *b*-AP15 (upsidedown triangles), DHA (open squares) in μM, Epo (closed squares) in nM × 100, WR (open circles) in nM × 10. Dotted line represents background (fluorescence of sample without Shield-1). Data is representative of at least five independent experiments. Additional details in Supplementary Fig. 2

Next, we examined the consequences of DHA exposure on global ubiquitination. Exposure of trophozoites to DHA for 90 min (Fig. 2a; Supplementary Fig. 2a) or mid-rings to DHA for 3 h (Supplementary Fig. 2b), resulted in a concentration-dependent increase in polyubiquitinated proteins, consistent with damage to proteins and overwhelming of the protein stress response. By contrast, exposure to chloroquine had little effect on polyubiquitination levels (Supplementary Fig. 2c). A similar build-up of polyubiquitinated proteins was induced by the proteasome inhibitor, epoxomicin (3 h) (Fig. 2b, Supplementary Fig. 3a, b.). This accumulation of ubiquitinated proteins appears to be toxic to parasites, as exposure to *b*-AP15, an inhibitor of the deubiquitinases that remove ubiquitin from substrates before degradation^24, 25^, or RA190, an inhibitor of the ubiquitin receptor^26^, both caused a build-up of ubiquitinated proteins (Fig. 2b, Supplementary Fig. 3a, b) and led to parasite death (Supplementary Fig. 2d).

### DHA treatment inhibits proteasome function

The rapid accumulation of ubiquitinated proteins in DHA-treated parasites led us to consider the possibility that DHA inhibits the activity of the proteasome. Increasing concentrations of DHA and epoxomicin decreased proteasome activity (as monitored using the proteasome-GLO system^27^) (Fig. 2c). Additionally, when extracts of treated parasites were subjected to native gel electrophoresis and incubated with a fluorogenic peptide substrate^28^, a significant decrease in 26S activity was evident, upon even a 60-min exposure to DHA (Fig. 2d, e), while a lethal dose of WR99210 (see kill curve in Supplementary Fig. 1c) did not inhibit activity (Fig. 2d, e). This effect did not result from a decrease in the abundance of the proteasome (as indicated by Western blotting; Supplementary Fig 2e), suggesting that DHA targets proteasome function, rather than causing proteasome turnover. The loss of proteasome activity may occur indirectly via clogging of the proteasome with ubiquitinated proteins, or directly via protein damage or dissociation of subunits, in a manner similar to that for oxidative damage of the mammalian 26S proteasome^29^, and consistent with a study showing that an ART derivative becomes cross-linked to a *P. falciparum* proteasome subunit^8^.

To further examine the in vivo activity of the proteasome in DHA-treated parasites we used transfectants expressing GFP fused to a destabilisation domain (DD)^30^ (Fig. 2f), which is targeted for degradation by the proteasome in the absence of the protective ligand, Shield-1. Inhibitors of proteasome-mediated degradation (epoxomicin, *b*-AP15 or RA190) prevented GFP-DD degradation in the absence of Shield-1 (Fig. 2g, Supplementary Fig. 2f). Similarly, treatment with DHA, but not WR99210, caused a build-up of full-length protein (Fig. 2g), confirming that DHA disrupts proteasome-dependent degradation.

### DHA treatment causes a build-up of damaged proteins

We used the fluorescence signal from GFP-DD to investigate protein unfolding (or misfolding or damage). In the absence of Shield-1, or upon treatment with WR99210, fluorescence was low but not abolished, consistent with degradation of part of the population of the fluorescent protein (Fig. 2h, Supplementary Fig. 2g). Treatment with Shield-1 increased the fluorescence signal, indicating an increase in full-length, properly folded GFP-DD. Treatment with epoxomicin, *b*-AP15 or RA190 also increased GFP-DD fluorescence (Fig. 2h, Supplementary Fig. 2g), indicating that the GFP remains folded when the proteasome is inactivated by these compounds. By contrast, upon DHA exposure, the GFP-DD fluorescence was reduced to below background (minus Shield-1) levels (Fig. 2h, Supplementary Fig 2g), consistent with protein unfolding/damage. Taken together, our findings show that DHA induces protein damage and prevents degradation via the proteasome, leading to an accumulation of damaged, polyubiquitinated proteins.

### Inhibiting polyubiquitination antagonises DHA activity

**Fig. 3:**
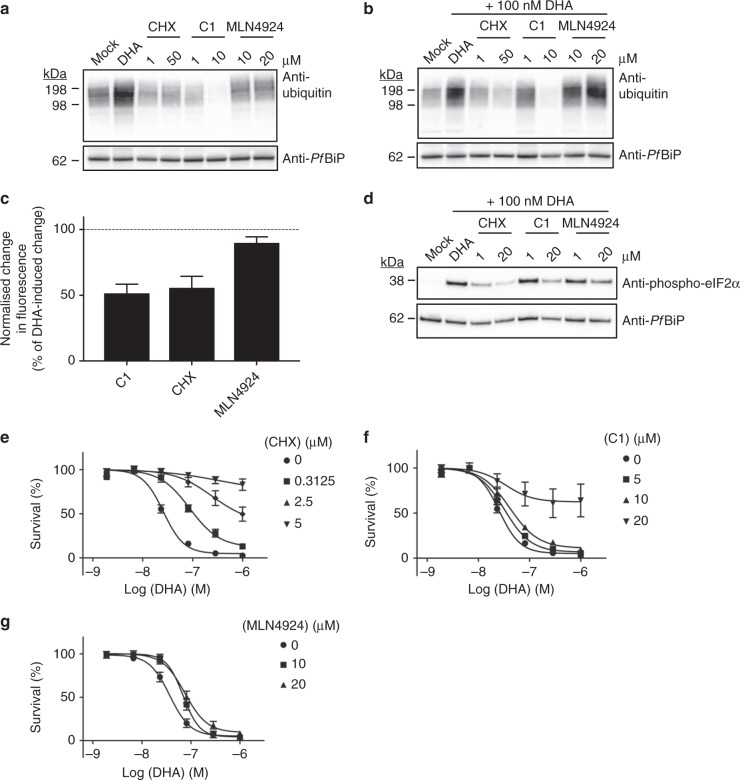
Compounds that inhibit protein polyubiquitination and protein damage antagonise the activity of DHA. **a**, **b** Trophozoites (3D7) were subjected to the indicated treatment for 6 h before analysis of ubiquitinated proteins. Samples treated with MLN4924 were incubated for an additional 3 h prior to DHA treatment. Blots are representative of 3–4 independent experiments (see Supplementary Fig. 3 for biological replicates). **c** Normalised maximum change in GFP fluorescence, measured by flow cytometry after GFP-DD trophozoites were incubated with 20 μM of the indicated compounds for 3 h, prior to exposure to DHA for 3 h. Dotted line represents 100%, the maximum decrease in fluorescence with DHA treatment. Error bars represent s.e.m. (*n* = 5 (C1), *n* = 4 (CHX, MLN4924)). **d** Trophozoites (Cam3.II_rev) were treated with 0.1% DMSO (mock), or indicated compounds for 3 h, followed by 3 h with DHA before lysates were probed for phosphorylated-eIF2α. Blot is representative of three independent experiments. Loading control, *Pf*BiP. **e**–**g** Ring stage cultures (Cam3.II_rev) were treated with vehicle (circles) or 0.3125 μM (squares), 2.5 μM (upright triangles) or 5 μM (upsidedown triangles) CHX (**e**) or 5 μM (squares), 10 μM (upright triangles) or 20 μM (upsidedown triangles) C1 (**f**) or 10 μM (squares) or 20 μM (upright triangles) MLN4924 (**g**) for 3 h, followed by a further 3 h with DHA. Following drug wash-out, parasitemia was assessed after 72 h. Error bars represent s.e.m. (*n* *=* 3). Additional details in Supplementary Figs. 4–6

To investigate further the nature of the toxic events leading to parasite killing, we examined different strategies for preventing the accumulation of proteasome substrates. CHX arrests protein translation, a state that protects mammalian cells from the lethality induced by proteasome inhibition^31^. We found that treatment of trophozoites with a pulse of CHX (up to 20 μM for 6 h) had no effect on viability (Supplementary Fig. 4a). Exposure of trophozoites to CHX decreased the base-line level of polyubiquitinated proteins (Fig. 3a, Supplementary Fig. 3c, d). Similarly, exposure to a combined pulse of CHX and DHA markedly reduced the DHA-mediated build-up of polyubiquitinated proteins (Fig. 3b, Supplementary Fig. 3e–f), and decreased DHA-mediated unfolding/damage of GFP-DD (Fig. 3c, Supplementary Fig. 4b). Importantly, CHX co-treatment strongly inhibited DHA-mediated eIF2α phosphorylation (Fig. 3d) and strongly antagonised DHA-mediated killing (Fig. 3e, Supplementary Fig. 4c, d), confirming that accumulation of unfolded, polyubiquitinated proteins is a key toxic event initiated by DHA treatment.

Compound 1 (5′-O-sulphamoyl-N(6)-[(1S)−2,3-dihydro-1H-inden-1-yl]-adenosine), hereafter referred to as C1, is a pan-inhibitor of ubiquitin-activating enzymes (E1), which depletes intracellular pools of activated ubiquitin^32^. Treatment of trophozoites with a pulse of C1 (6 h up to 20 μM) had no effect on parasite viability (Supplementary Fig. 5a). However, it decreased the base-line level of polyubiquitinated proteins (Fig. 3a, Supplementary Fig. 3c, d) and increased the GFP-DD fluorescence in the absence of Shield-1 (Supplementary Fig. 5b), consistent with accumulation of proteasome substrate that is not recognised/degraded as it is not ubiquitinated. Additionally, C1 treatment reduced DHA-mediated accumulation of polyubiquitinated proteins to below background levels (Fig. 3b, Supplementary Fig. 3e–f) and strongly inhibited the DHA-mediated unfolding/damage of GFP-DD (Fig. 3c, Supplementary Fig. 4b). Accordingly, C1 reduced the DHA-induced build-up of phosphorylation of eIF2α (Fig. 3d) and strongly antagonised DHA-mediated killing (Fig. 3f, Supplementary Fig. 5c, d). C1 also antagonized the build-up of polyubiquitinated proteins and killing induced by epoxomicin (Supplementary Fig. 5e, f), but did not prevent DHA-mediated damage to proteasome function (Supplementary Fig. 5g).

Cullin ring E3 ubiquitin ligases are covalently modified with NEDD8, which regulates their role in ubiquitin-dependent proteolysis^33^. Treatment with a pulse of MLN4924, an inhibitor of mammalian NEDD8 activating enzyme^32^, did not inhibit growth of parasites when applied alone (Supplementary Fig. 6a). It did not cause a marked reduction in polyubiquitinated proteins (Fig. 3a, b, Supplementary Fig. 3c–f), nor did it inhibit DHA-mediated unfolding/ damage to GFP-DD (Fig. 3c, Supplementary Fig. 4b). MLN4924 weakly decreased eIF2α phosphorylation (Fig. 3d), and mildly, though significantly, antagonised killing (Fig. 3g, Supplementary Fig. 6b, c).

## Discussion

Consistent with recent reports showing that ARTs induce widespread cellular damage^6–8^, our data indicate that DHA causes protein damage/unfolding and inhibits folding of newly synthesised proteins. This likely leads to global perturbation of cell function and viability, compromising protein functionality^8^ and inducing protein aggregation^34^.

We show that the DHA-initiated proteostatic stress induces eIF2α phosphorylation via activation of PK4, the *Plasmodium* homologue of mammalian PERK. By analogy with PERK, PK4 is likely an integral membrane protein in the ER with an N-terminal sensor of unfolded proteins protruding into the ER lumen (the sequence has one strongly predicted transmembrane domain and several other putative transmembrane helices). Upon accumulation of damaged proteins, the cytoplasmic C-terminal catalytic domain is activated and phosphorylates eIF2α. Indeed, we show that DHA treatment leads to prolonged eIF2α phosphorylation and attenuation (but not arrest) of protein synthesis, consistent with stress-induced, eIF2α-mediated disruption of translation. The decreased protein translation initiated by DHA likely underpins reports of growth retardation^35^ and quiescence^36^ upon exposure to ARTs.

Indeed, while this manuscript was under review, another study^37^ showed that DHA treatment leads to an increase in eIF2α phosphorylation and repression of translation and that overexpression of a PK4 dominant-negative mutant blocks translational arrest and enhances the ability of DHA to kill parasites. This work provides complementary evidence for an important contribution of the stress response to the killing mechanism.

We propose that an unresolved ER stress response underpins DHA-mediated toxicity, compounded by DHA-mediated (direct or indirect) inhibition of proteasome function, which would prevent removal of damaged proteins, compromise amino acid homeostasis^31^ and inhibit processing of important cellular factors^38^, leading to eventual proteostasis network collapse^39^. Importantly, we show that following short duration exposure to DHA, damage to specific protein targets occurs, but is not, in and of itself, the toxic event. That is, we show that in the presence of a potent inhibitor of ubiquitin activation (C1), DHA treatment still leads to decreased proteasome function, but parasite killing is largely avoided. Similarly, inhibition of translation by CHX, which as expected decreases protein ubiquitination, strongly antagonises the action of DHA. The antagonism of DHA-mediated killing by an inhibitor of NEDD8 activation (MLN4924) suggests the involvement of a cullin E3-mediated ubiquitin ligation event, although additional work is needed to confirm the involvement of NEDD8.

**Fig. 4:**
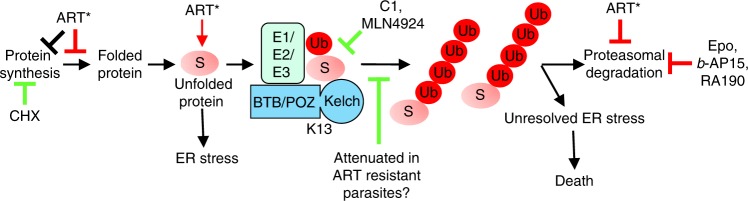
Model of DHA-mediated killing of *P. falciparum*. Activated ART (ART*) initiates a multi-pronged assault on protein homeostasis by damaging and unfolding proteins, preventing folding of newly synthesised proteins and inhibiting the proteasome (toxicity indicated with red bar). Activation of the ER Stress response, attenuates translation. Prolonged activation of the ER Stress response, and accumulation of toxic polyubiquitinated substrates (S) eventually leads to cell death. Other proteasome inhibitors also cause accumulation of polyubiquitinated proteins and parasite killing (Epoxomicin (Epo), *b*-AP15, and RA190). Inhibition of the E1/E2/E3 ubiquitin machinery (C1 or MLN4924) or ablation of protein synthesis (cycloheximide, CHX), prevents accumulation of polyubiquitinated proteins and enables cell survival (rescue indicated with green bar). In ART resistant parasites, defective presentation of proteins for polyubiquitination, may enable cell survival

Taken together our results lead us to propose that DHA packs a double whammy, damaging and unfolding proteins and inhibiting proteasome function, with the consequent build-up of ubiquitinated proteins as the key event that leads to cell death, likely via the unresolved ER stress pathway^35^. As illustrated in Fig. 4, this work defines the critical events in the mechanism of killing by ARTs.

Our work provides insights into the possible role of K13 in ART action. K13 shares sequence similarity with BTB/Kelch substrate adaptors that facilitate ubiquitin ligation by cullin-3 ligases^9^, such as the putative *P. falciparum* cullin-3 (PF3D7_0629800). Human cullin-3 works with ~70 different BTB/Kelch adaptors to ubiquitinate different substrates. By contrast, K13 is the only BTB/Kelch protein encoded by *P. falciparum*, consistent with it playing a major role in targeting a substantive protein sub-set to the proteasome. We propose that K13 is an adaptor that plays a critical role in presenting damaged proteins for ubiquitination, leading either directly or indirectly to enhanced ubiquitination levels, and that the consequent build-up of polyubiquitinated proteins clogs the proteasome^40^, eventually leading to cell death (Fig. 4). The enhanced survival of *K13* mutants may be due to a defect in presenting damaged proteins for polyubiquitinatation. Increased expression of chaperones and proteostasis pathway proteins^41^, and an increased basal level phosphorylation of eIF2α in early ring stage parasites^37^ likely also contribute to survival.

Our work identifies the ubiquitin-proteasome system as a point of critical vulnerability in *P. falciparum*, suggesting a number of potential targets, including the proteasome itself^38^ and the deubiquitinating enzymes that feed proteins to it^42^. A recent saturation mutagenesis showed that 54 of the 72 genes in the proteasome degradation pathway can be classified as essential and that single-insertion mutants of an ART sensitivity cluster of genes showed increased sensitivity to the proteasome inhibitor, Bortezomib^43^. It is of particular interest that mutations in the deubiquitinase, UBP1, have been observed in ART-pressured parasites^12^, and that UBP1 mutations are also associated with decreased ACT effectiveness in Africa^44^ and South East Asia^45^. Mutations in different components of the ubiquitin-proteasome pathway may enhance ART resistance by helping to reduce accumulation of polyubiquitinated proteins. Obversely, targeting components of the ubiquitin-proteasome system provides a means of overcoming resistance-inducing mutations, thus maintaining the impact of control programs.

## Methods

### Materials

Dihydroartemisinin (DHA), DTT, DMSO, CHX, WR99210 (WR), D-(+)-GlcN were from Sigma-Aldrich; SYTO-61 was from Life Technologies; Protease Inhibitor Cocktail (PIC) was from Roche; epoxomicin was from Sapphire Bioscience or Sigma; *b*-AP15 was from Calbiochem; RA190 was from Calbiochem; Compound 1 (C1) was a kind gift of Dr Larry Dick, Takeda Pharmaceuticals, and MLN4924 was from MedChemExpress. Shield-1 ligand was kindly provided by Dr Dean Goodman and Prof Geoff McFadden, University of Melbourne, or purchased from Cheminpharma.

### Culturing and cell lines

*P. falciparum* parasites used in this study were propagated in O+ human RBCs (Australian Red Cross Blood Service) in RPMI-1640, supplemented with GlutaMAX^TM^, 25 mM HEPES (ThermoFisher), 5% (v/v) human serum (Australian Red Cross Blood Service), 0.25% (w/v) AlbuMAX II (Life Technologies), 10 μM d-glucose, 22 μg mL^−1^ gentamycin, and 0.5 mM hypoxanthine, and incubated at 37 °C in an atmosphere of 1% O_2_, 5% CO_2_ and 94% N_2_. Cultures were monitored by Giemsa staining of methanol-fixed blood smears. Culture media was replaced at least every 48 h and parasitemia was maintained below 10% to ensure health of cultures.

*P. falciparum* strains used in this study were: 3D7 laboratory strain, Cam3.II^rev^ (*K13*^*WT*^)^10^ and a GFP-DD reporter strain generated with plasmid pEF-GFP-HA-DD24 (ref. ^46^), kindly provided by Dr Mauro F Azevedo and Dr Paul Gilson (Burnet Institute). *eIK1* and *eIK2* knock-out lines were kindly provided by Prof Christian Doerig (Monash University)^20^. *PK4-HA-glmS* ribozyme-inducible knockdown parasites were generated by PCR amplification of the ~850 bp region preceding the 3′ end of *pk4* (PF3D7_0628200, gDNA isolated from 3D7). PCR products were ligated into p*GlmS*-*3HA* to generate final targeting vectors^22, 47^. Cell lines were verified to be mycoplasma free.

### Analysis of cell lysates by Western blotting

Trophozoite parasite cultures at 5% haematocrit and 5% parasitemia were subjected to the drug treatments indicated in the figure legends at 37 °C. Note that concentrations used do not result in loss of binding of a nucleic acid probe within the 90 min, but would be lethal if the parasites were maintained in the presence of drug through to the next asexual cycle. For anti-ubiquitin assays, cell pellets were washed with ice-cold PBS supplemented with complete mini EDTA-free PIC (Roche) and 20 mM *N*-ethylmaleimide. RBC cytoplasmic contents were released with 0.15% (w/v) saponin and parasite pellets were washed with PBS. For experiments using GFP-DD parasites, trophozoite-stage parasites were isolated with 0.05% w/v saponin and pellets were washed in PBS, supplemented with EDTA-free PIC (Roche). Parasite pellets were solubilised in reducing SDS-PAGE sample buffer, boiled at 95 °C for 5 min, resolved by SDS-PAGE on a 4–12% Bis-Tris acrylamide gel (Life Technologies) using MES or MOPS running buffer and transferred to nitrocellulose membrane (iBlot; Life Technologies). Membranes were blocked with 5% (w/v) skim milk for 1 h at room temperature and probed with primary antibody overnight at 4 °C, followed by secondary antibody for 1 h at room temperature. Primary antibodies: rabbit anti-ubiquitin (Dako-Z0458; 1:1000 or Cell Signaling Technology #3933; 1:1000); rabbit anti-phospho-eIF2α (Cell Signaling Technology-119A11; 1:1000); mouse anti-eIF2α (Cell Signaling Technology-L57A5; 1:1000); rabbit anti-*Pf*GAPDH (ref. ^48^; 1:1000); polyclonal mouse anti-*Pf*BiP was generated using recombinant *Pf*BiP at the WEHI Antibody Services (1:1000); mouse anti-GFP (Roche; 1:1000); mouse anti-HA (Sigma-H3663; 1:500); mouse anti-20S alpha 1-2-3-5-6-7 (Abcam-22674; 1:500). Secondary antibodies: goat anti-rabbit IgG-peroxidase (Sigma-Aldrich-A0545; 1:20,000), goat anti-mouse IgG-peroxidase (Chemicon-AP127P; 1:20,000). Chemiluminescence was detected using the Bio-Rad ChemiDoc^TM^ MP imaging system. Biological replicates of some blots are provided in Supplementary Figure 3. Uncropped versions of some blots are provided in Supplementary Figure 7.

### Protein synthesis assays

Trophozoite-stage cultures at 2% haematocrit and 10% parasitemia were enriched by magnet purification and allowed to recover at 37 °C for 1 h prior to experimentation. 1 × 10^7^ cells were subjected to the drug treatment indicated in the figure legend in the presence of ^3^H-tryptophan (20.1 mCi mmol^−1^ stock, Perkin Elmer) at 37 °C. Samples were washed with ice-cold PBS and the acid-insoluble radioactive protein products were precipitated in 30% (w/v) TCA. The precipitated protein pellet was washed once with 5% (w/v) TCA and once with MilliQ ddH_2_O. Precipitated proteins were solubilised by incubation in 1 M NaOH at 50 °C for 30 min. The mixture was decolourised by incubation in 12.5% (w/v) NaClO for 20 min at room temperature. Solubilised radiolabelled proteins were then diluted in MilliQ ddH_2_O and transferred to vials containing scintillation fluid for β-scintillation counting using a Tri-Carb 4810TR Liquid Scintillation Counter (Perkin Elmer). Data were processed and visualised using GraphPad Prism software (version 5). Values presented are normalised to mock (0.1% DMSO)-treated sample (100% ^3^H-Trp incorporation).

### Analysis of proteasome activity by proteasome-GLO system

Trophozoite-stage parasite cultures at 3% haematocrit and 4% parasitemia were subjected to the drug treatment indicated in the figure legend. RBC cytoplasmic components were released by incubation in 0.05% (w/v) saponin and parasite pellets were washed with ice-cold PBS three times. The parasite pellet was lysed in ice-cold water (pH 4.5) and the supernatant was cleared by centrifugation at 760×*g* for 10 min. One microgram cell lysate was mixed with an equal volume of the Proteasome-GLO (chymotrypsin-like) assay reagents (Bio-Rad). Luminance was measured using CLARIOstar microplate reader (BMG Labtech). Measurements were taken every 2 min for 3 h. Average readings between 60 and 90 min (after the signal reached equilibrium) were used for proteasome activity analysis. Values are normalised to mock (0.1% DMSO)-treated sample (100% Proteasome-GLO signal).

### Analysis of proteasome activity by native gel electrophoresis

Trophozoite-stage parasite cultures at 2.5% haematocrit and 5% parasitemia were enriched by magnet purification and subjected to the drug treatment indicated in the figure legend at 37 °C. Cell pellets were washed with ice-cold PBS and RBC cytoplasmic components released by incubation in 0.15% (w/v) saponin. The parasite pellet was solubilised in hypotonic lysis buffer (25 mM Tris-HCl, 10 mM MgCl_2_, 1 mM ATP, pH 7.4) on ice for 30 min. The supernatant was cleared by centrifugation at 13,000×*g* for 15 min and analysed for protein content by BCA assay (Pierce).

Samples were analysed for proteasome activity using an in-gel assay^49^. Fifty microgram cell lysate was mixed with native loading dye (250 mM Tris-HCl pH 7.4, 50% (v/v) glycerol, 0.0007% xylene cyanol) and loaded onto a 4% acrylamide gel. Electrophoresis was carried out at 130 V for 90 min at 4 °C. Following electrophoresis, the gel was incubated in developing buffer (50 mM Tris-HCl pH 7.4, 5 mM MgCl_2_, 1 mM ATP) supplemented with 100 μM suc-LLVY-AMC (Enzo Life Sciences) for 30 min at 37 °C, after which time the signal was captured under UV light using the Bio-Rad ChemiDoc^TM^ MP imaging system. All reagents contained 1 mM ATP to maintain proteasome activity.

For Coomassie staining, following UV imaging, the gel was washed three times in MilliQ ddH_2_O and stained with SimplyBlue^TM^ SafeStain (Invitrogen) according to the manufacturer’s instructions. Densitometric analysis was carried out using ImageJ software (Gel Analysis method). The integrated pixel intensity for each entire lane was corrected for background signal and normalised to the sum of the signal produced by all samples (100% proteasome activity). Brightness and contrast were not adjusted in the main figure.

### Measurement of GFP fluorescence using flow cytometry

Trophozoite-stage parasite cultures at 5% haematocrit and 5% parasitemia were subjected to the drug treatment indicated in the figure legends at 37 °C. Culture samples were stained with 2 μM SYTO-61^50^ and adjusted to 0.1% haematocrit in PBS. SYTO-61 and GFP fluorescence was recorded using the FACSCanto™ II cytometer (Becton Dickinson). Analysis was performed in FlowJo (version10). Parasites were gated based on the SYTO-61 signal, and parasite GFP fluorescence values reported are mean fluorescence values. Data were fit by nonlinear regression using GraphPad Prism Software (version 5).

### Assessment of parasite viability following drug treatment

Drug pulse assays were performed using established methods^13, 51–53^. Tightly synchronised parasites (0–2 h post invasion (h.p.i.) for ring stage or 24–27 h.p.i. for trophozoite stage) at 0.2% hematocrit and 1–2% parasitemia were subjected to the indicated drug pulse at 37 °C. Cells pellets were washed thoroughly to remove drug and returned to culture until parasites reached trophozoite stage of the following cycle. Cells were then stained with 2 μM SYTO-61 and their fluorescence measured by flow cytometry (FACSCanto™ II cytometer; Becton Dickinson). Data were gated and analysed using FlowJo software (version 7.6.5) to determine parasitemia of each sample. Parasitemia values were then graphed by nonlinear regression using GraphPad Prism software (version 5). Survival (%) represents the parasitemia normalised to untreated (100% survival) and kill-treated (0% survival) controls, where kill-treated refers to samples treated with 2 μM DHA for 48–72 h.

### Statistical analysis

Statistical significance between groups was determined using one-way ANOVA with Dunnett’s Multiple Comparison Test (Figs. 1c, 2e) or unpaired two-tailed *t*-test (Supplementary Fig. 6c) (with GraphPad Prism Software, version 5). *P* values were considered significant if *P* < 0.05. In all cases, *n*, refers to the number of biological replicates.

## Electronic supplementary material

Supplementary Information

## Data Availability

All data are available from the authors on request.
