# Supplementary material for: Artemisinin kills malaria parasites by damaging proteins and inhibiting the proteasome
Source: Nat Commun. 2018 Sep 18;9:3801. doi: 10.1038/s41467-018-06221-1 (PMC6143634; doi:10.1038/s41467-018-06221-1)
Supplement: Supplementary file 1 — Supplementary Information [file 41467_2018_6221_MOESM1_ESM.pdf]

Supplementary Materials for

Artemisinin kills malaria parasites by damaging proteins and inhibiting the proteasome

Bridgford et al.

This PDF file includes: Supplementary Figures 1 to 7

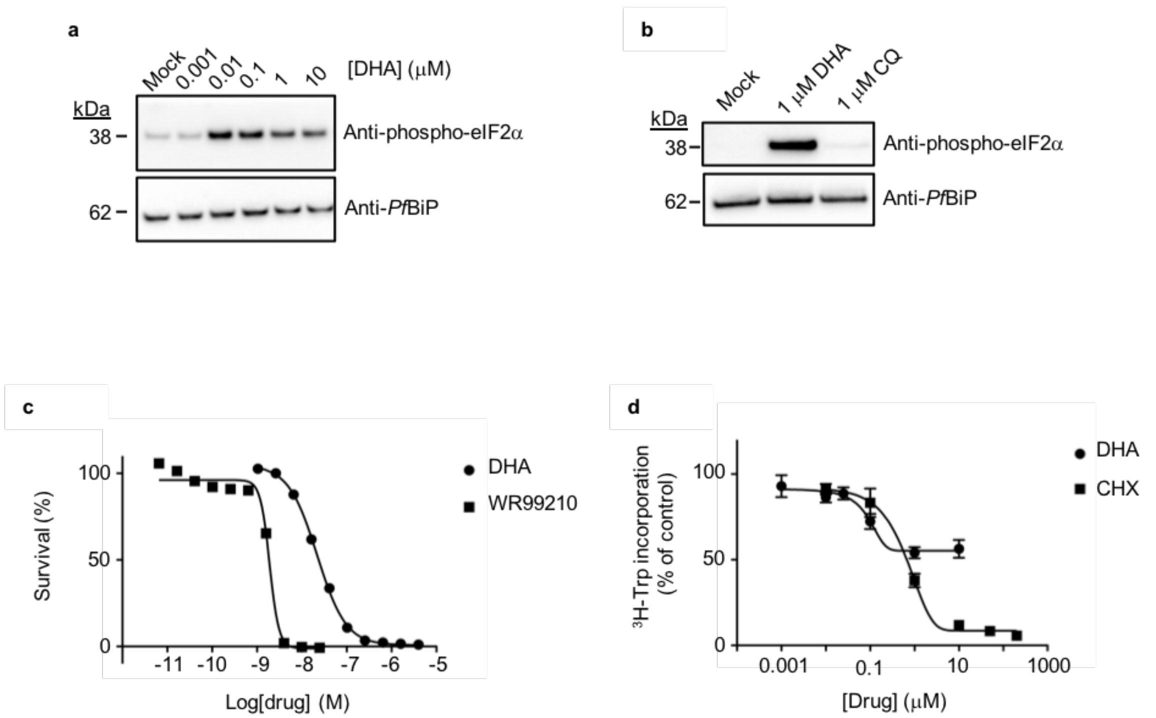

Supplementary Figure 1. Analysis of DHA and control drug-induced unfolded protein response, translation arrest and killing. (**a**) Mid-ring stage-infected RBCs (Cam3.II_rev) were treated with 0.1% DMSO (mock) or DHA for 3 h and harvested before lysates were subjected to Western blot analysis and membranes probed for phosphorylated-eIF2α. Blot is representative of three independent experiments. (**b**) Trophozoite-infected RBCs (3D7) were treated with 0.1% DMSO (mock) or DHA or chloroquine (CQ) for 60 min and harvested before lysates were subjected to Western blot analysis and membranes probed for phosphorylated-eIF2α. Blot is representative of two independent experiments. (**c**) Trophozoite-stage parasites (3D7) were treated with DHA (circles) or WR99210 (squares) for 1 h before the drugs were washed out. Parasites were returned to culture and parasitemia was measured in the following trophozoite stage. Data are representative of two independent experiments. (**d**) Protein synthesis was measured in trophozoite stage parasites (~30 h post invasion; 3D7, 5 x 10^7 cells) that were exposed to increasing concentrations of DHA (circles) or cycloheximide (CHX, squares) for 30 min (in RPMI without AlbuMax at 37°C) and then incubated in AlbuMax-free RPMI containing the same drug concentration plus ^{3}H-tryptophan for 1 h (37°C). Radiolabelled proteins were precipitated, solubilised, and scintillation counted. Error bars represent s.e.m. (*n* = 3).

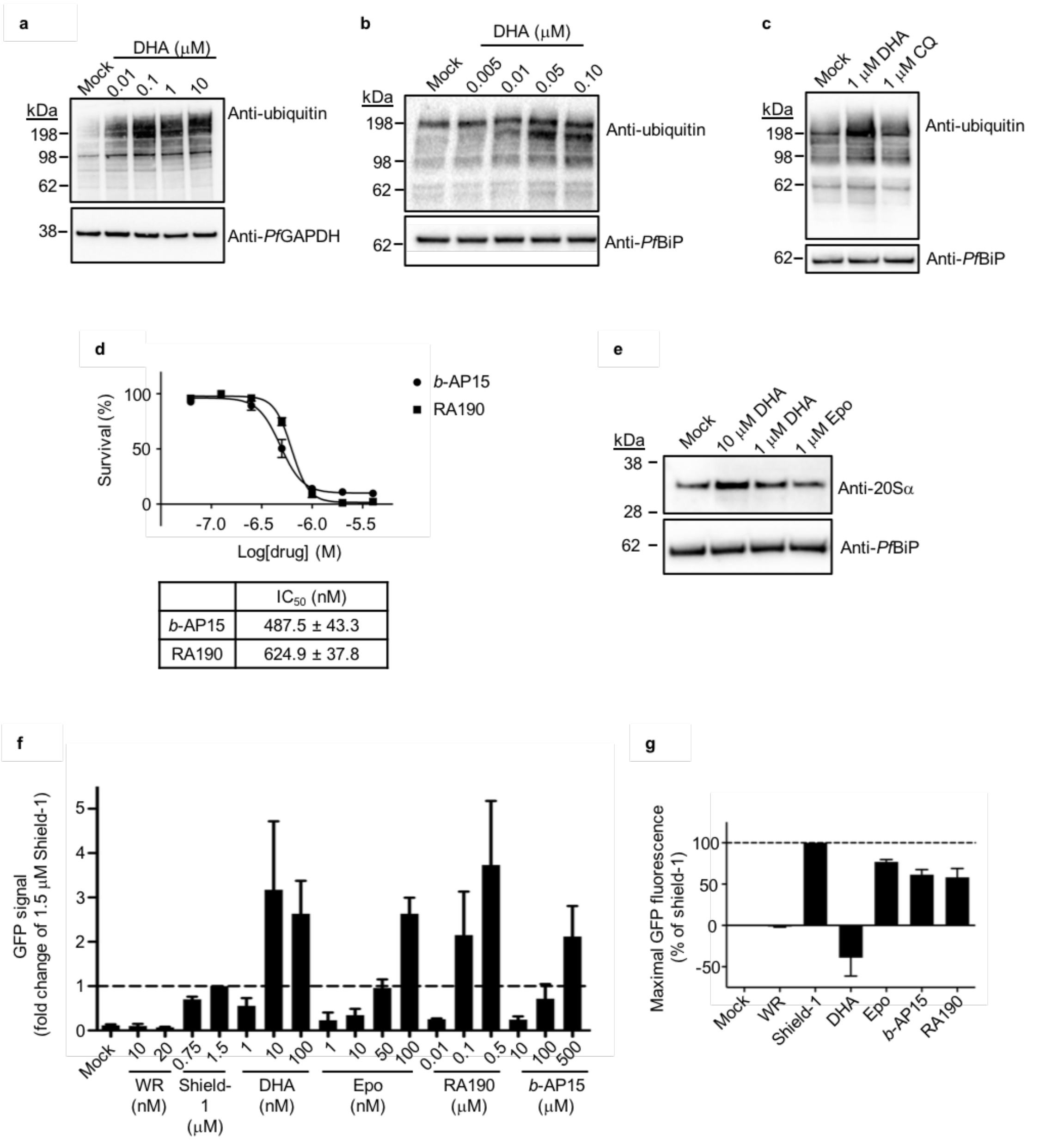

Supplementary Figure 2. Accumulation of polyubiquitinated proteins, protein unfolding and toxicity of inhibitors of ubiquitin-dependent proteasome activity. (**a-c**) Infected RBCs (Cam3.II_rev trophozoites (**a**) or Cam3.II_rev ring stage 15% parasitemia (**b**) or 3D7 trophozoites (**c**)) were treated with 0.1% DMSO (mock) or DHA for 90 min (**a**) or 3 h (**b**) or DHA or chloroquine (CQ) for 60 min (**c**) before lysates were subjected to Western blot analysis. Loading controls, *Pf*GAPDH (a) or *Pf*BiP (b, c). (**d**) Ring stage parasite cultures (0-3 h post invasion; Cam3.II_rev) were treated with various concentrations of *b*-AP15 (circles) or RA190 (squares) for 3 h at 37ºC. The drug was washed out and parasites were returned to culture for 72 h before assessment of parasitemia by flow cytometry. Error bars represent s.e.m. (*n* = 3). (**e**) Western blot analysis of 20S proteasome α sub-units (1-3, 5-7) after treatment of trophozoite-stage parasites (Cam3.II_rev) with 0.1% DMSO (mock), DHA or epoxomicin for 1 h. Blot is

representative of three independent experiments. Loading control, *Pf*BiP. (**f**) Grouped quantitation of Western blot data corresponding to Fig. 2g. The GFP signal was normalised to *Pf*BiP signal and expressed as fold change relative to 1.5 μM Shield-1. Error bars represent s.e.m. (*n* = 7 (DMSO), *n* = 6 (DHA), *n* = 5 (Shield-1 1.5 μM), *n* = 4 (Epo 10 nM, 100 nM), *n* = 3 (WR, Shield-1 0.75 μM; RA190; *b*-AP15), *n* = 2 (Epo 1 nM, 50 nM). (**g**) Grouped quantitation of GFP fluorescence data corresponding to Fig. 2h. Dotted line indicates mean maximal fluorescence at 1/1.5 μM Shield-1. Data represent the mean % maximal fluorescence (at two highest concentrations tested) relative to Shield-1 (100%) and mock (0.1% DMSO)-treated samples from 5-13 independent experiments including Fig. 2h. Error bars represent s.e.m. (*n* = 5 (*b*-AP15, RA190, WR), *n* = 13 (Shield-1, DHA, Epo)).

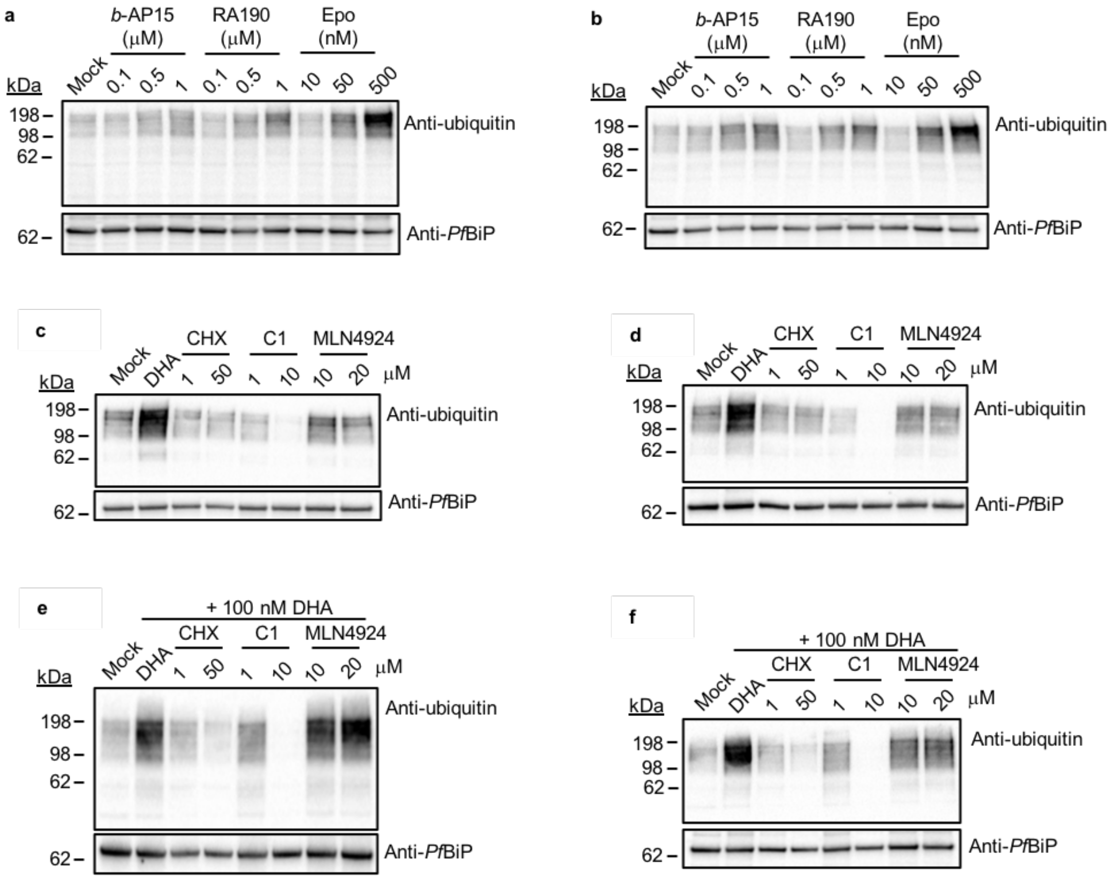

Supplementary Figure 3. Biological replicates of blots presented in the main figures. **(a, b)** Biological replicates of blot prsented in Fig. 2b. Trophozoites (3D7) were incubated with 0.1% DMSO (mock) or DHA (90 min) or *b*-AP15, RA190 or Epo (epoxomicin) (3 h) and extracts were probed for ubiquitinated proteins. **(c-f)** Biological replicates of blots presented in Fig. 3a (c, d) and Fig. 3b (e-f). Trophozoites (3D7) were subjected to the indicated treatment for 6 h before analysis of ubiquitinated proteins. Samples treated with MLN4924 were incubated for an additional 3 h prior to DHA treatment.

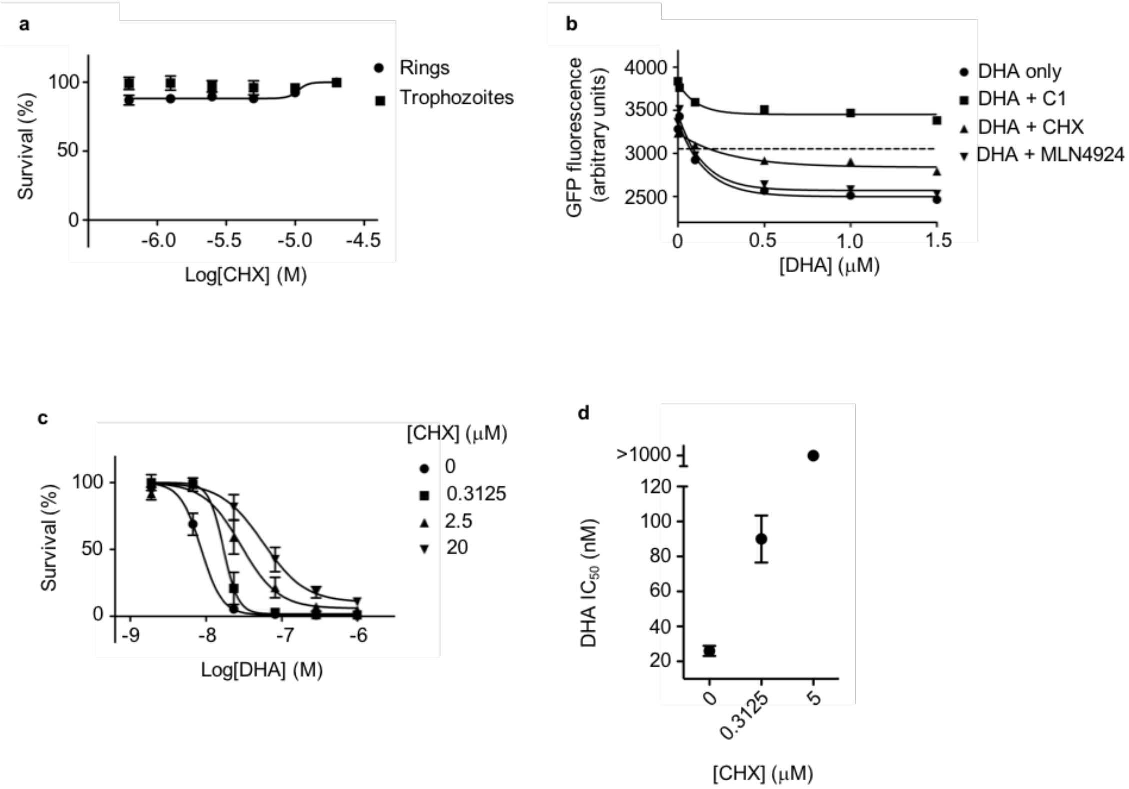

Supplementary Figure 4. Cycloheximide (CHX) antagonises DHA-mediated build-up of polyubiquitinated proteins, protein unfolding and killing. (**a**) Ring stage (0-2 h post invasion, circles) or trophozoite stage (24-26 h post invasion, squares) parasites (Cam3.II_rev) were subjected to a 6 h pulse with CHX and parasitemia assessed in the following cycle. (**b**) Flow cytometry signal after GFP-DD trophozoites (in a 3D7 background) were incubated with 20 μM of C1 (squares), CHX (upright triangles), MLN4924 (upsidedown triangles) or vehicle (circles) for 3 h, prior to exposure to DHA for 3 h (raw data corresponding to Fig. 3c). Dotted line represents background (fluorescence of sample without Shield-1). Data is representative of at least four independent experiments. (**c**) Trophozoite stage parasites (24-26 h post invasion; Cam3.II_rev) were incubated with 0.3135 μM (squares), 2.5 μM (upright triangles) or 20 μM (upsidedown triangles) CHX or vehicle (circles) for 3 h prior to a 3 h DHA pulse, and parasitemia was assessed in the following cycle. (**d**) Analysis of CHX antagonism of DHA activity in a ring stage survival assay (corresponding to data in Fig. 3e). In all cases, error bars represent s.e.m. (n = 3).

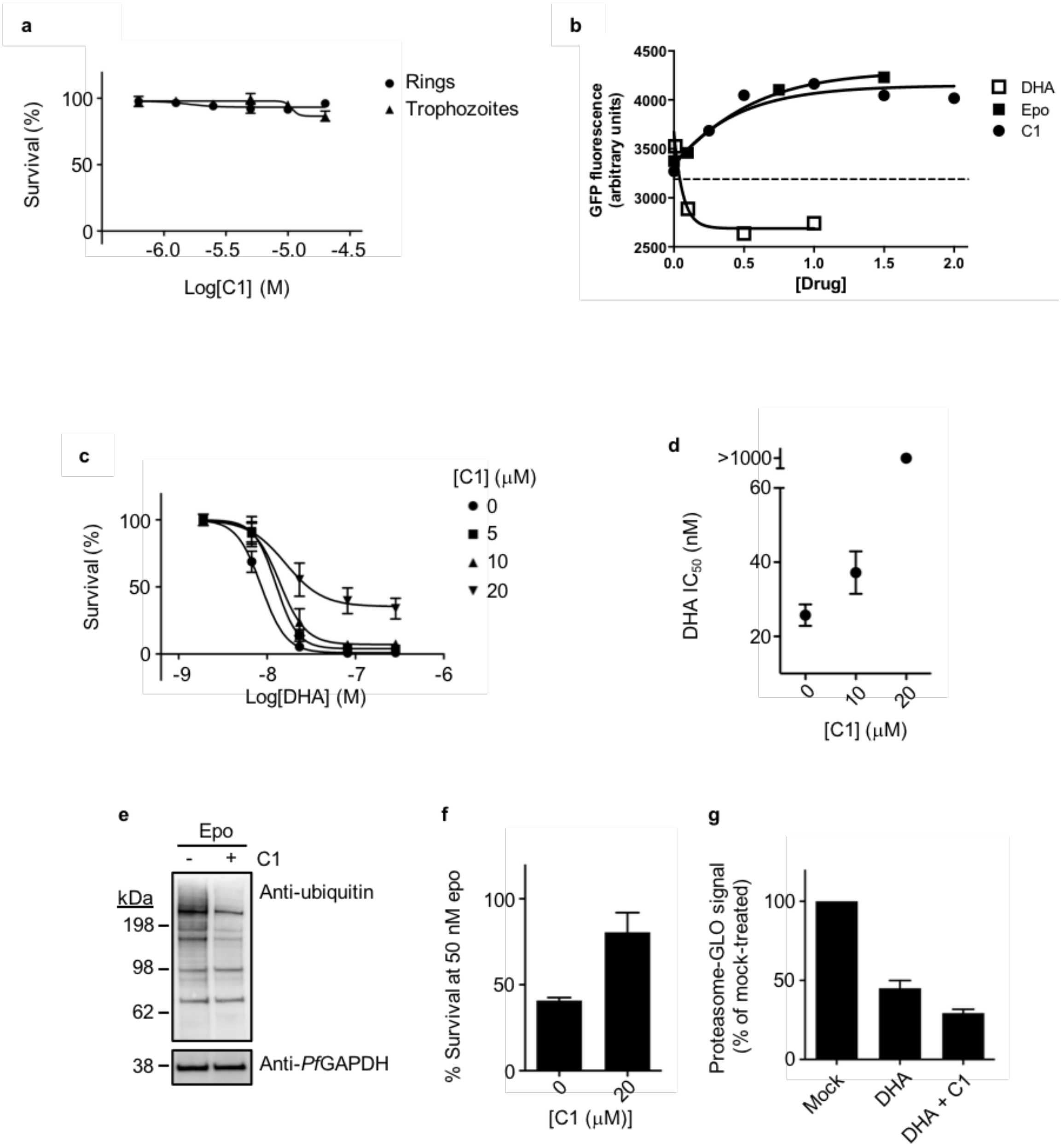

Supplementary Figure 5. C1 antagonises DHA and epoxomicin but does not prevent functional inhibition of the proteasome. (**a**) Ring stage (0-2 h post invasion, circles) or trophozoite stage (24-26 h post invasion, triangles) parasites (Cam3.II_rev) were subjected to a 6 h pulse with C1 and parasitemia assessed in the following cycle. **(b)** GFP-DD transfectants (in a 3D7 background) were maintained in Shield-1 for 24 h, before wash-out. Trophozoites were exposed to DHA (open squares), Epo (closed squares) or C1 (circles) for 4 h, and GFP fluorescence measured by flow cytometry. Dotted line represents background (fluorescence of sample without Shield-1). Data are representative of two independent experiments. (**c**) Trophozoite stage parasites (24-26 h post invasion; Cam3.II_rev) were incubated with 5 μM (squares), 10 μM (upright triangles) or 20 μM (upsidedown trianlges) C1 or vehicle (circles) for 3 h prior to a 3 h DHA pulse, and

parasitemia was assessed in the following cycle. (**d**) Analysis of C1 antagonism of DHA activity in a ring stage survival assay (corresponding to data in Fig. 3f). (**e**) Trophozoite stage parasites (Cam3.II_rev) were treated with 400 nM epoxomicin (Epo) with or without 10 μM C1 for 3 h at 37ºC and lysates assessed by Western blot for ubiquitinated proteins. Loading control, *Pf*GAPDH. (**f**) Ring stage parasites (0-2 h post invasion; Cam3.II_rev) in cell culture were treated with 50 nM epoxomicin (Epo) with or without 20 μM C1 for 3 h at 37ºC. The compounds were washed out and parasites were returned to culture for 72 h before assessment of parasitemia by flow cytometry. Error bars represent s.e.m. (n = 2). (**g**) Trophozoite stage parasites (Cam3.II_rev) were pre-incubated with or without 20 μM C1 for 3 h, then incubated in the presence of 1 μM DHA for 3 h. Proteasome activity in cell lysates was measured using the proteasome-GLO system. In all cases error bars represent s.e.m. (n = 3, except where otherwise stated).

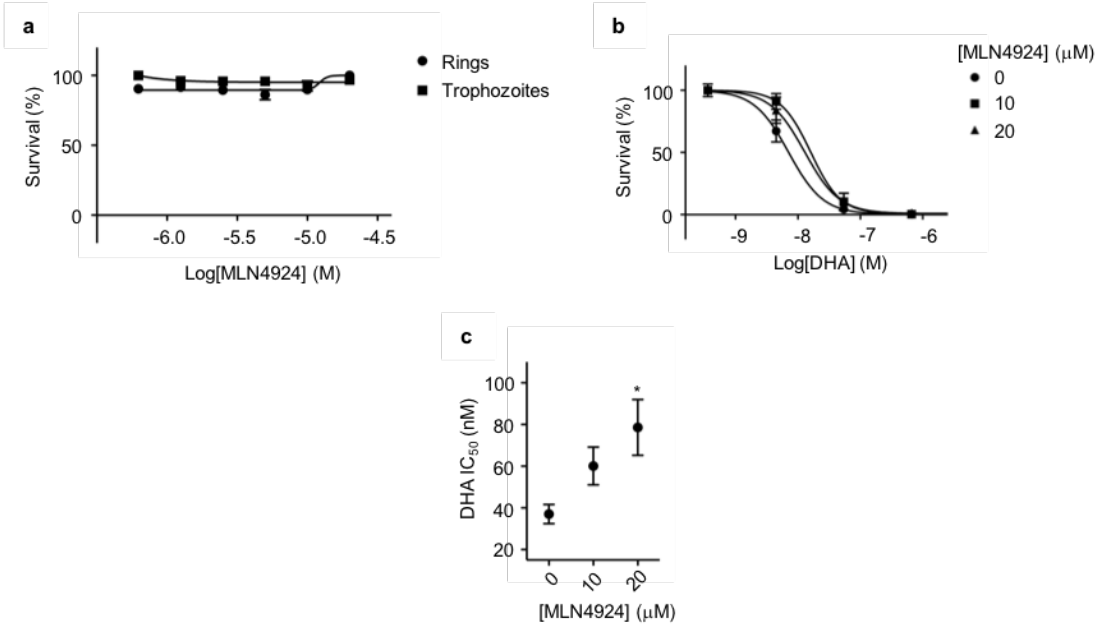

Supplementary Figure 6. MLN4924 antagonises DHA-mediated killing. (**a**) Ring stage (0-2 h post invasion, circles) or trophozoite stage (24-26 h post invasion, squares) parasites (Cam3.II_rev) were subjected to a 6 h pulse with MLN4924 and parasitemia assessed in the following cycle. (**b**) Trophozoite stage parasites (24-26 h post invasion; Cam3.II_rev) were incubated with 10 μM (squares) or 20 μM (triangles) MLN4924 or vehicle (circles) for 3 h prior to a 3 h DHA pulse, and parasitemia was assessed in the following cycle. (**c**) Analysis of MLN4924 antagonism of DHA activity in a ring stage survival assay (corresponding to data in Fig. 3g). *$P = 0.042$ between 20 μM MLN4924 and no MLN4924 treated samples, using two-tailed unpaired *t*-test. In all cases, error bars represent s.e.m. (n = 3)

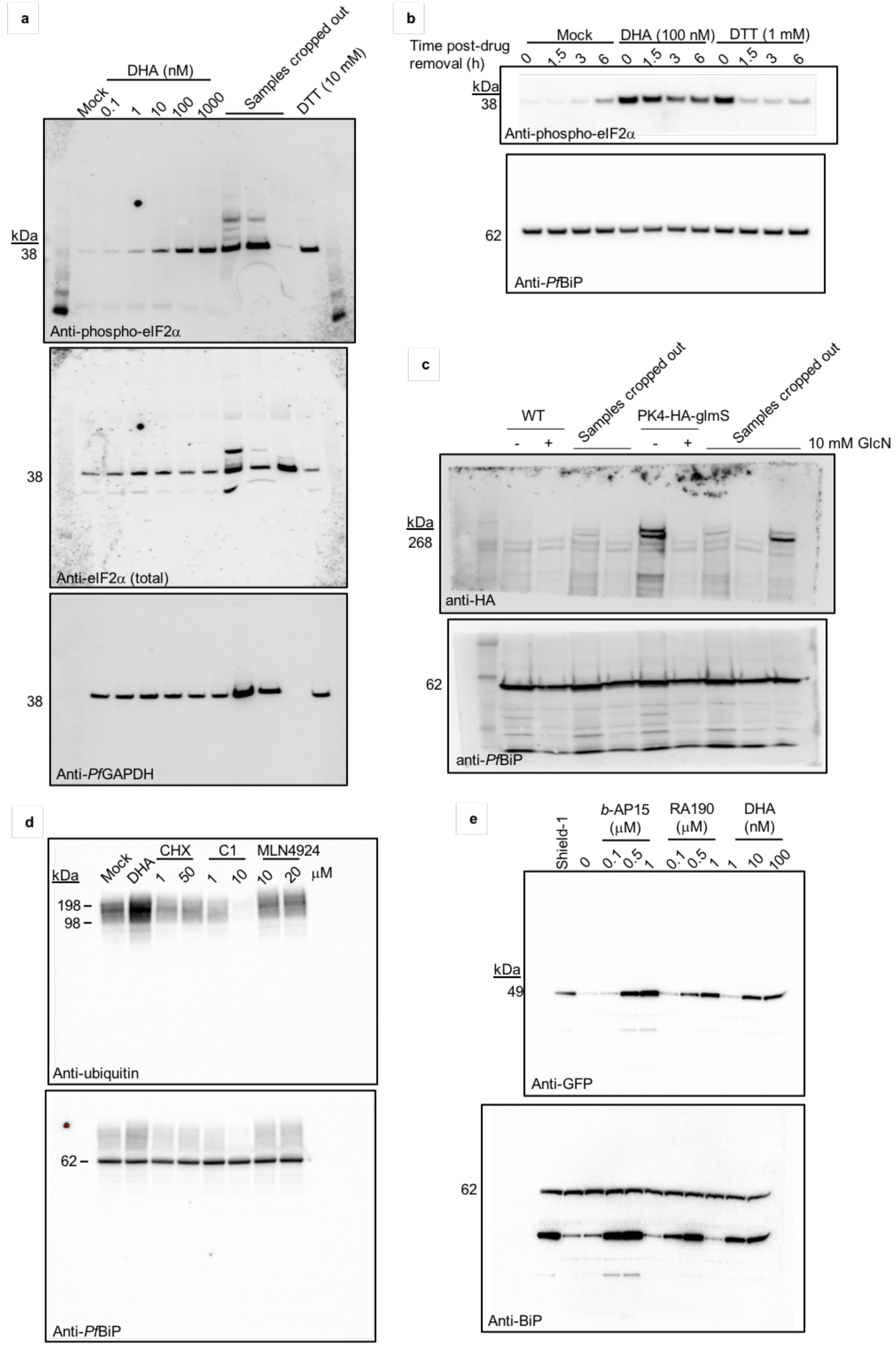
a
Mock
DHA (nM)
0.1
1
10
100
1000
Samples cropped out
DTT (10 mM)
kDa
38
Anti-phospho-eIF2α
38
Anti-eIF2α (total)
38
Anti-PfGAPDH
b
Time post-drug removal (h)
Mock
DHA (100 nM)
DTT (1 mM)
0 1.5 3 6 0 1.5 3 6 0 1.5 3 6
kDa
38
Anti-phospho-eIF2α
62
Anti-PfBiP
c
WT
Samples cropped out
PK4-HA-glmS
Samples cropped out
- + - +
10 mM GlcN
kDa
268
anti-HA
62
anti-PfBiP
d
Mock
DHA
CHX
1
50
C1
1
10
MLN4924
10
20
μM
kDa
198
98
Anti-ubiquitin
62
Anti-PfBiP
e
Shield-1
b-AP15 (μM)
RA190 (μM)
DHA (nM)
0 0.1 0.5 1 0.1 0.5 1 1 10 100
kDa
49
Anti-GFP
62
Anti-BiP

Supplementary Figure 7. Uncropped versions of Western blots. (a) Uncropped blots corresponding to those presented in Fig. 1a. Blot was originally probed with rabbit anti-phospho-eIF2α (top), stripped and reprobed with mouse anti-eIF2α (total, middle) and then stripped again and reporbed with rabbit anti-*Pf*GAPDH (bottom). **(b)** Uncropped blots corresponding to Fig. 1b. The blot was cut into two sections prior to probing: 28-49 kDa, which was probed with anti-phospho-eIF2α (top); and 49-98 kDa, which was probed with anti-*Pf*BiP (bottom). **(c)** Uncropped blots corresponding to those presented in Fig 1e. The blot was cut into two sections prior to probing: >98 kDa, which was probed with anti-HA (top); and <98 kDa, which was probed with anti-*Pf*BiP. **(d)** Uncropped blots corresponding to those presented in Fig. 3a. The blot was initially probed with anti-ubiquitin (top) and then with anti-*Pf*BiP (bottom). **(e)** Uncropped blots corresponding to those presented in Fig. 2g (right panel). The blot was initially probed with mouse anti-GFP (top), then reblocked and probed with rabbit anti-*Pf*BiP (bottom).
